# A hyperbolic decay of subjective probability of obtaining delayed rewards

**DOI:** 10.1186/1744-9081-3-52

**Published:** 2007-09-25

**Authors:** Taiki Takahashi, Koki Ikeda, Toshikazu Hasegawa

**Affiliations:** 1 Department of Life Sciences, Unit of Cognitive and Behavioral Sciences, School of Arts and Sciences, The University of Tokyo, 21 COE office, 3-8-1 Komaba, Meguro-ku, Tokyo, 153-8902, Japan

## Abstract

**Background:**

Hyperbolic discounting of delayed and probabilistic outcomes has drawn attention in psychopharmacology and neuroeconomics. Sozou's evolutionary theory proposed that hyperbolic delay discounting may be totally attributable to aversion to a decrease in subjective probability of obtaining delayed rewards (SP) which follows a hyperbolic decay function. However, to date, no empirical study examined the hypothesis, although this investigation is important for elucidating the roles of impatience, precaution, and uncertainty aversion in delay discounting processes.

**Methods:**

In order to (i) determine the functional form of the relation between delay until receipt and SP, and (ii) examine whether delay discounting is attributable to a decrease in SP, we assessed the subjects' SP and their delay and probability discounting. We examined the fitness of hyperbolic and exponential functions to the assessed SP, and relations between the SP, and delay/probability discounting, and subjective-probability discounting for delayed rewards.

**Results:**

The results demonstrated (a) SP decayed hyperbolically as delay increases, (b) a decay of SP was associated with delay discounting, and (c) subjective-probability discounting did not significantly correlate with delay discounting.

**Conclusion:**

Our results demonstrated (i) hyperbolic decay of SP is related to delay discounting, and (ii) delay discounting is, however, not attributable to precautious foresight in intertemporal choice. Further, a novel parameter of pure time preference is proposed.

## Background

### Delay discounting

People prefer an immediate reward to a delayed one (referred to as "delay discounting"). Psychopharmacological and neuroeconomic studies have demonstrated that drug-dependence, attention-deficit/hyperactivity disorders (ADHDs) are associated with greater delay discounting (referred to as "impulsivity" in intertemporal choice [1-10]). Impulsivity in delay discounting processes may consist of two types of psychological processes: (i) aversion to waiting (or inability to wait) for the delayed rewards (referred to as "pure time preference" or "impatience" in intertemporal choice [6]) and (ii) aversion to uncertainty associated with delay [9,11,12]. If delay discounting occurs due only to uncertainty aversion [13], strong discounting of delayed rewards ("impulsivity" in intertemporal choice) should not be regarded as impairment in self-control (i.e., impatience), but as a forward-looking and risk-aversive tendency (precautious uncertainty aversion). On the contrary, if impulsive delay discounting is associated with impatience; i.e., simple aversion to waiting for delayed rewards (also referred to as "pure time preference"), subjects with greater delay discounting (e.g., substance abusers and ADHD patients) may have impaired self-control (e.g., impatience or impulsivity in psychiatry's sense, see Appendix I) [7,14]. Although dissociating delay discounting processes into these subcomponents is important for a better understanding of impulsive behaviors and establishing better medical treatments, to date, no study has successfully achieved the dissociation, partly due to a lack of good theoretical frameworks in the previous empirical investigations. By utilizing an evolutionary theory proposed by Sozou [13], our present study examined the relationship between delay discounting, uncertainty aversion as a subcomponent of delay discounting (i.e., "subjective-probability discounting" for delayed rewards, which psychologically corresponds to "precautious uncertainty aversion" in intertemporal choice) and pure uncertainty aversion (probability discounting). Because a recent study reported that nicotine addicts evaluated delayed rewards as less certain than non-smokers [9], the present study may help understand impulsivity and inconsistency in intertemporal choice (see Appendix I for a distinction between impulsivity and inconsistency) by drug-dependent patients and substance misusers.

Standard economic theory has assumed that a discount rate is independent of *D *(dynamic consistency, see Appendix I for details) [6], leading to the exponential discount function (see Appendix II for mathematical characteristics of exponential discounting):

*V*_*D *_= *Aexp(-k*_*d*_*D)*

where *V*_*D *_is the subjectively discounted value of the reward at delay *D*, *A *is the undiscounted value of the reward = *V*_*D*_(*D *= 0), *D *is the delay to the receipt of the reward, and *k*_*d *_is a free parameter [6]. The larger *k*_*d *_becomes, the more rapidly a subject discounts the delayed reward (more impulsive intertemporal choice). However, empirical studies in humans and non-human animals reported that delay discounting is better described by the hyperbolic function (see Appendix II for mathematical characteristics of hyperbolic discounting) [1-10]:

*V*_*D *_= *A/(1+k*_*d*_*D)*

with the same notations as in Equation 1. Again, a larger *k*_*d *_value corresponds to more rapid discounting. Therefore, in hyperbolic discounting, subjects underestimate their future impulsivity, resulting in preference reversal as time passes (also referred to as dynamic inconsistency, see Appendix I) [6,7]. Because normative decision theory and microeconomics state that hyperbolic discounting is not rational (see Appendix I and II), previous studies have investigated why human and animal intertemporal choice is hyperbolic [6,7,13,15,16]. However, we still do not have the final answer to this question. It is important to understand psychological processes underlying hyperbolic discounting, because problematic behaviors in temporal discounting (e.g., loss of self-control in drug-dependent patients and substance misusers) are associated with inconsistency, rather than impulsivity, in intertemporal choice (see Appendix I) [7]. Therefore, studies in the nascent field of neuroeconomics attempted to elucidate neural correlates of inconsistency in intertemporal choice (hyperbolicity in temporal discounting) [15,16], in addition to impulsivity in intertemporal choice.

### Sozou's hypothesis on hyperbolic delay discounting

One of the accounts for delay discounting is that delayed rewards are discounted because more delayed rewards are more uncertain. In order to explain hyperbolic discounting for delayed rewards, an evolutionary theorist Sozou proposed the following two assumptions [13]:

(A) subjective probability of obtaining delayed rewards decays hyperbolically,

(B) the subjective value of a delayed reward equals a statistical expected value in terms of subjective probability.

It is to be noted that assumption B excludes the psychological process of aversion to waiting for a delayed reward (impatience) from candidate accounts for (impulsivity in) delay discounting.

Let us briefly see the mathematical characteristics of Sozou's hypothesis (see Appendix III for details). Because an exponential decay function [13]:

*SP(D) = exp(-k*_*sp*_*D)*,

where *k*_*sp *_indicates a decay rate of SP as delay D increases, cannot explain hyperbolic delay discounting, Sozou has proposed that SP(D) follows the hyperbolic decay function [11]:

*SP(D) = 1/(1+k*_*sp*_*D)*,

in order to derive the hyperbolic delay-discounting function. Note that larger *k*_*sp *_indicates a more rapid decay of SP as delay increases; i.e., a high degree of "precaution" in intertemporal choice. Note also that *k*_*sp *_does not measure subject's aversion to uncertainty associated with delay, but simply measure subject's estimation of potential risk factors in the future.

To date, no study examined the validity of the key assumption (A). One of the objectives of the present study is to directly examine whether SP(D) follows the exponential or hyperbolic function, in order to test the Sozou's assumption A. Moreover, studies in probability discounting (devaluation of uncertain rewards) imply that assumption (B) may not always be correct, because the subjective value of an uncertain reward does not exactly equal the statistical expected value (explained below).

### Probability discounting

Subjects discount the value of uncertain rewards as the probability of receiving the rewards decreases [3,8,11,17]. This behavioral tendency has been referred to as "probability discounting" (psychologically, also referred to as "uncertainty aversion"). Rachlin et al [17] have proposed the following exponential and hyperbolic probability-discounting functions:

*V*_*p *_= *Aexp(-k*_*p*_*O)*

and

*V*_*p *_= *A/(1+k*_*p*_*O)*,

where *V*_*p *_is a subjective discounted value of a probabilistic reward, *A *is the value when *p *= 1, *O *is the odds against = (1/*p*)-1 (proportional to an average waiting time in a repeated gambling), and *k*_*p *_is the probability discount rate. *k*_*p *_indicates the degree to which one discounts the uncertain reward. Several studies found that hyperbolic probability discounting function (Equation 6) fits the behavioral data better than the exponential discount function (Equation 5). Recently, a psychopharmacologist Bickel's group invented a framework combining delay and probability discounting [11]. We therefore adopted *k*_*p *_as subject's uncertainty aversion parameter (note that larger *k*_*p *_corresponds to strong uncertainty aversion).

### Discounting of delayed rewards due to decrease in subjective probability

Let us again consider discounting of delayed rewards occurring due to aversion to uncertainty associated with delay (i.e., "precautious uncertainty aversion" in intertemporal choice), rather than due to impatience. The "precautious uncertainty aversion" in intertemporal choice is a result of both "precaution" (i.e., estimation of potential risks in the future) and "uncertainty aversion" (i.e., aversion to the estimated risks in the future). It is to be noted that, as can be seen from the hyperbolic probability-discounting function (Equation 6), the subjective value of an uncertain reward in Rachlin's model is equal to a statistical expected value if and only if *k*_*p *_= 1 (because *A/(*1+1 × *[(*1*/p)-*1*]) = pA*). Therefore, Sozou's assumption B may exactly be true only when *k*_*p *_= 1. In contrast, when *k*_*p *_is not equal to 1, the assumption (B) should be modified as:

(B') the subjective value of a delayed reward equals the subjective value of an uncertain reward in terms of subjective probability.

Hence, if the assumption (B') (in other words, delay discounting is totally attributable to precautious uncertainty aversion; i.e., hyperbolic subjective-probability discounting due to a decrease in SP as delay increases) is correct, the subjective value of a delayed reward *V*_*D *_should follow the (hyperbolic) subjective-probability discounting function:

*V*_*D *_*(SP) = A/(1+k*_*spd*_*O*_*sp*_*) = A/(*1*+k*_*spd*_*[(*1*/SP)-*1*])*,

where SP is a subjective probability of obtaining the reward at each delay D, subjective odds-against *O*_*sp *_is defined as *(*1*/SP)*-1 and the parameter *k*_*spd *_indicates the degree of "precautious uncertainty aversion"; in other words, the degree to which a subject discount the delayed reward due, solely, to aversion to subjective uncertainty associated with delay (not due to aversion to waiting; i.e., impatience). The independent variable of the subjective-probability discounting function is SP at delay D, not delay D *per se*. Therefore, larger *k*_*spd *_corresponds to the degree of a forward-looking but precautious and risk-aversive tendency.

### Objectives of the present study

This study had three main goals: (i) to determine the functional form of subjective probability of obtaining delayed rewards (SP) as a function of delay, (ii) to examine the relationship between the decay rate of SP(D) (i.e., *k*_*sp *_which indicates the degree of "precaution" in intertemporal choice) and rates of delay and probability discounting (*k*_*d *_and *k*_*p*_, which indicate the degrees of impulsivity in intertemporal choice and uncertainty aversion in probabilistic choice, respectively), (iii) to examine whether subjective-probability discounting for delayed rewards (i.e., *k*_*spd*_, which indicates the degree of "precautious uncertainty aversion" in intertemporal choice) is associated with delay discounting (i.e., *k*_*d*_). If *k*_*spd *_(precautious uncertainty aversion) and *k*_*d *_(impulsivity, which consists of both "impatience" and "precautious uncertainty aversion") are strongly correlated, impulsivity in intertemporal choice (i.e., delay discounting) is predominantly be explained by precautious uncertainty aversion (i.e., subjective-probability discounting due to uncertainty associated with delay), rather than impatience.

In order to assess subjects' SP, we developed a subjective probability questionnaire (SPQ) explained later. It should be noted that, if Sozou's hypothesis is perfectly correct, (i) SP as a function of delay may be hyperbolic rather than exponential (from Sozou's assumption A), and (ii) *k*_*sp *_and *k*_*d *_are positively correlated (predicted from Sozou's hypothesis), and (iii) *k*_*spd *_and *k*_*d *_may be positively correlated (from assumption B'). Alternatively, if a delay in intertemporal choice, via distinct psychological processes, induces both "impatience" and "precaution" (i.e., a decrease in SP), *k*_*sp *_and *k*_*d *_may be significantly correlated, but no significant correlation may be observed between *k*_*spd *_and *k*_*d*_. Because Sozou's and Rachlin's theories are irrelevant to the effects of the sign (i.e., gain or loss) and the magnitude of delayed outcomes on discounting behavior [6,18], we did not examine these effects in the present study.

## Methods

### Participants

Thirty-one college students (age: 19.4 ± 0.3) were recruited (13 men, 18 women) at the University of Tokyo. Past or present smokers or substance abusers were excluded from participation in this experiment.

### Delay and probability discounting tasks

First, participants performed the delay/probability discounting task. It is to be noted that we have previously developed and utilized exactly the same Japanese version of the discounting task [14], and the paper-and-pencil discounting tasks were originally developed by Bickel's group [19].

Participants were requested to choose alternatives based solely on their free will, as if choices were about real money [14,19]. Instructions for each questionnaire were written on the top of each page of the questionnaire, and expressed the temporal distance of delay (1 week, 2 weeks, 1 month, 6 month, 1 year, 5 years, 25 years, each page included each delay and delays were presented in this order) in the delay condition, and the probability for uncertain reward (95%, 90%, 70%, 50%, 30%, 10%, 5%, each page included each probability, and probabilities were presented in this order) in the probability condition. Two columns of hypothetical money amounts were listed below the instructions. The right column (standard amount) contained 40 rows of a fixed magnitude of money (= 1,000 yen). The left column (adjusting amount) listed ascending or descending magnitudes of money in 2.5% increments (= 1000 yen × 0.025 = 25 yen) of the alternative in the right column. Participants were instructed to choose between the two alternatives in each row of the questionnaire. Furthermore, as in the Bickel and colleagues' discounting task, participants were directed to attend to the directions on the top of each page (containing each delay or probability) of the questionnaire, as the temporal distance would change over the course of experiment. Thus, subjects chose between delayed-standard amount and immediate-adjusted amount of money in the delay condition, and between uncertain-standard amount and certain-adjusted amount of money in the probability condition. The order of the descending and ascending conditions was counterbalanced.

The indifference points of delay and the probability tasks were defined as the means of the largest adjusting value in which the standard alternative was preferred and the smallest adjusting value in which the adjusting alternative was preferred. Next, the mean of the indifference point in ascending and descending adjusting amounts were calculated for the delay and probability conditions in each participant.

### Subjective probability questionnaire (SPQ)

In addition, we asked the subjects to estimate the subjective probability of obtaining a reward (SP) with delays corresponding to the delay discounting task. In order to develop a questionnaire for the assessment of SP (SPQ), we modified the questionnaire in Reynolds' studies [9,12], in which participants were asked to rate their psychological certainties for delayed rewards on a 1–10 point rating scale ("delay-discounting certainty questionnaire", DDCQ) [9,12]. SPQ was employed because (a) the DDCQ is not appropriate for assessing the value of participants' subjective probability of obtaining a delayed reward, and (b) the objective of the present study was to determine the functional form of SP(D), which is crucial for testing Sozou's theory [13]. Participants were instructed as follows (in Japanese):

You had chosen alternatives between immediate and delayed rewards. Please again imagine as if the questions were about real monetary rewards.

If you had chosen the money delayed by 1 week, [the delays were different for each question], how were you sure you would actually get that money in 1 week? Please answer your subjective probability of obtaining the delayed reward in the unit of percentage.

The same question was repeated with corresponding delays (1 week, 2 weeks, 1 month, 6 months, 1 year, 5 years, 25 years, in this order, similarly to previous studies [9,12]). As in Patak and Reynolds' studies [9,12], participants were given minimal instruction on completing SPQ, except to "answer each question based on the intertemporal choice questions just completed". The discounting tasks and SPQ procedures were conducted in the order of delay discounting, probability discounting, and SPQ. The rationale for employing this order is that (i) the behavioral data in the delay discounting task was the most important, (ii) conducting SPQ (which make participants to associate delay with probability) before the probability discounting task may artificially strengthen the subjective association between probability and delay in the probability discounting task. It is also to be noted that the orders of the delay and probability discounting tasks did not affect indifference points in our previous study [8].

### Data analysis

Indifference points for individual and group median data were obtained in order to compare the goodness-of-fit between the exponential and the hyperbolic models in delay and probability discounting. The data of one subject did not show the consistency for defining the indifference point (i.e., because there were two switching points at the same delay, the indifference point at the delay could not be defined) in delay discounting and therefore were excluded from further analysis [8].

Subjective probabilities (SPs) of obtaining delayed rewards were also obtained and the goodness-of-fit was examined, for the hyperbolic and the exponential decay models, at individual and group levels. For each discounting/decay model, we performed a nonlinear regression in order to estimate a free parameter in the model. Next, we, with the estimated best-fit parameters, calculated the Akaike information criterion with small sample correction (AIC_c_, a second order AIC) for exponential and hyperbolic functions in delay, probability, and subjective-probability discounting, and a subjective probability decay, respectively [20]. It should be noted that the definition of the second order AIC is: AIC_c _: = - 2 *Ln *([Residual Sum of Squares]/*n*) +2*Kn/(n-K*-1*)*, where *Ln *is a natural log, *K *is the number of estimated parameters in the model, *n *is the sample size [21]. We have utilized AIC_c _in the present study because *n/K *< 40 [20,21]. Note that the smaller AIC_c _becomes, the better a model approximates the data [21]. Although AIC_c_, in general, indicates a tradeoff between overfitting and poor fitting [21], AIC_c _indicates a goodness-of-fit in the present study. The reason is that all the models (i.e., exponential and hyperbolic discounting/decay models) contain the same number of a free parameter (= 1) in the present study. For statistical comparisons of AIC_c_s at the individual level between models, we utilized t-tests [20], because the Kolmogorov-Smirnov tests did not revealed a significant deviation from the normal distribution (*p*_*s *_> 0.05) [20]. It is to be noticed that when R-square values were employed, essentially the same results were obtained.

After confirming that hyperbolic models better fit the data than exponential models for all behavioral data (i.e., delay discounting, probability discounting, SP(D)(i.e., SP at each delay D), and subjective-probability discounting, see Results), we examined relationship between *k *parameters (i.e., *k*_*d*_*, k*_*p*_, *k*_*sp *_and *k*_*spd*_) in the hyperbolic models (not in the exponential models). Because the *k *parameters did not distribute normally (Kolmogorov-Smirnov test, *p*_*s *_< 0.05), correlations between *k *parameters (i.e., *k*_*d*_*, k*_*p*_, *k*_*sp *_and *k*_*spd *_in the hyperbolic models) were assessed by nonparametric (Spearman's rank-order) correlation tests. It is important to note that, if *k*_*spd *_is significantly correlated with *k*_*d*_, delay discounting may mainly be attributable to precautious uncertainty aversion (subjective-probability discounting due to a decrease in SP as delay increases) [13].

All statistical procedures were conducted with R statistical language (the R project for statistical computing). The alpha level was set at 0.05 throughout (for multiple comparisons, Bonferoni's correction was utilized).

## Results

### Relation between delay and probability discounting rates

**Figure 1:**
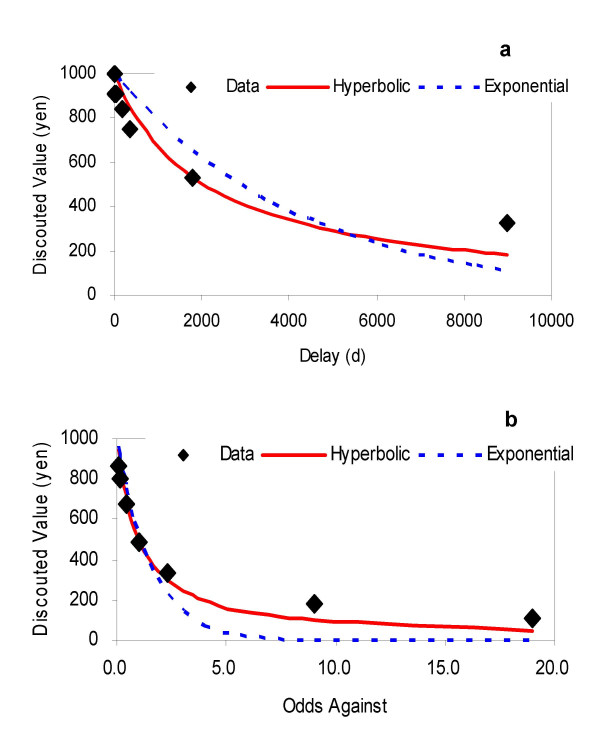
The solid (red) and dotted (blue) curves are the hyperbolic and exponential functions respectively, best fitted to the experimental data in delay and probability discounting tasks (a-b) (see Methods). Fig. 1a: Vertical axis indicates group median of indifference point in delay discounting task (discounted present value) and horizontal axis indicates delay (days). Data points are represented by the black diamonds. Fig. 1b: Vertical axis indicates group median of indifference point in probability discounting task (certainty equivalent discounted value) and horizontal axis indicates odds-against = (1/probability)-1 (an average waiting time in Rachlin's *virtually*-*repeated *gambling [17]). Note that hyperbolic models (red) better fit than exponential models (blue) (see Table 1 for AIC_c _for each model).

Delay and probability discounting functions fit to behavioral data of group median indifference points are shown in Fig. 1a and 1b. Fig. 1a shows the group median of each indifference points in the delay discounting. Fig. 1b shows the probability discounting with a horizontal axis as an odds against. AIC_c_s for group data showed better fit (smaller values) for hyperbolic functions (Table 1), consistent with previous studies [1-11]. When AIC_c_s for individual discounting data were calculated, hyperbolic discount functions also significantly better fit individual data than exponential functions (*t*-tests, *p*_*s *_< 0.01, for all comparisons), also suggesting that subjects discounted delayed and probabilistic rewards hyperbolically.

**Table 1:** AIC_c _(Akaike Information Criterion with small sample correction) for exponential and hyperbolic functions in delay and probability discounting, and SP (subjective probability of obtaining delayed rewards)

	Exponential	Hyperbolic
Delay discounting	92.33	86.49
Probability discounting	90.59	82.33
SP (subjective probability)	-6.7	- 14.37

Hyperbolic functions better fit behavioral data in delay and probability discounting and SP. Note that smaller AIC_c _indicates better fitting.

Each individual's *k*_*d *_and *k*_*p *_were estimated by Equation 2 and 10 for delay and probability discounting, respectively (Table 2). Spearman's correlation coefficients (*rho*) between two parameters were shown in Table 3. There was no significant correlation between *k*_*d *_and *k*_*p *_(*p*_*s *_> 0.1), in line with recent studies reporting that delay and probability discount rates are at best weakly correlated [3,8,22].

**Table 2:** Each subject's delay (*k*_*d*_) and probability (*k*_*p*_) discount rates and decay rate *k*_*sp *_of subjective probability of obtaining delayed rewards (SP).

Subject's ID	*k*_*d*_	*k*_*p*_	*k*_*sp*_	Subject's ID	*k*_*d*_	*k*_*p*_	*k*_*sp*_
1	0.000620	1.3047	*n.a*.	16	0.001757	0.9972	0.012409
2	0.000320	1.5601	0.025945	17	0.000455	1.0612	0.027894
3	0.002862	1.3140	0.007099	18	0.000928	1.0243	0.000631
4	0.000385	1.1339	*n.a*.	19	*n.a*.	0.4244	0.000116
5	0.004214	1.1543	0.025158	20	0.000144	1.4166	*n.a*.
6	0.000388	0.3495	0.022878	21	0.000053	0.6046	0.000333
7	0.000645	1.6229	0.002207	22	0.000378	0.5443	0.013914
8	0.004837	2.8802	0.002156	23	0.000224	24.9100	0.225340
9	0.001377	0.5892	0.000737	24	0.000115	0.8915	0.000031
10	0.021098	1.4429	0.000059	25	0.000170	1.3478	0.000002
11	0.008886	1.1245	0.017660	26	0.001646	3.4975	0.000026
12	0.035444	1.5373	0.028255	27	0.054930	0.9969	0.027064
13	0.000131	2.1120	0.000030	28	0.000052	0.7414	0.000347
14	0.000035	0.6274	0.000038	29	0.004808	0.0337	0.312700
15	0.000000062	0.5930	0.000640	30	0.001757	1.0714	0.015360

				Median	0.000489	1.0170	0.001395

Note that all *k *parameters were estimated with hyperbolic functions (not with exponential functions). *n.a*. indicates failure in estimating parameters in nonlinear regression [20].

**Table 3:** Spearman's correlations between the discount rates in delay discounting (*k*_*d*_), probability discounting (*k*_*p*_), and decay rate of SP (*k*_*sp*_)

	Probability discounting (*k*_*p*_)	Decay rate of SP (*k*_*sp*_)
Delay discounting (*k*_*d*_)	*rho*(27) = 0.20, *p *= 0.30	*rho*(24) = 0.467*, *p *= 0.016
Probability discounting (*k*_*p*_)		*rho*(25) = -0.01, *p *= 0.976

A significant correlation between delay discounting and decay rate of SP was observed (Spearman's rank-order correlation, *:*p *< 0.05). Note that a (hyperbolic) decay rate of SP (subjective probability of obtaining a delayed reward) as a function of delay (*k*_*sp*_) is defined in SP(D) = 1/(1+*k*_*sp*_D) (see [13]).

### Comparison of exponential and hyperbolic SP decay functions

**Figure 2:**
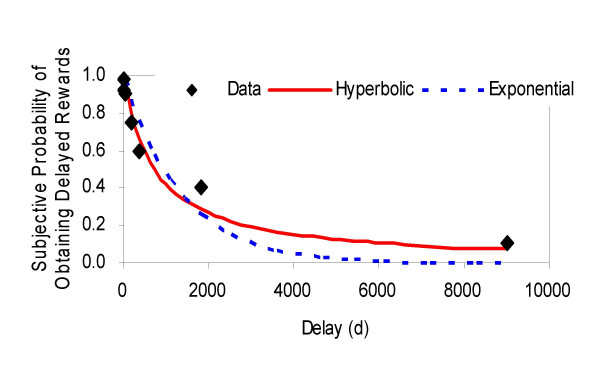
Vertical axis indicates group median of SP(D) (i.e., subjective probability of obtaining a reward at delay D) and horizontal axis indicates delay (days). The solid (red) and dotted (blue) curves are the hyperbolic and exponential functions respectively, best fitted to the SP(D) obtained in SPQ (see Methods). Note that hyperbolic decay model (red) proposed by Sozou [13] better fit data than exponential decay model (blue) (see Table 1 for AIC_c _for each model).

We compared the fitness of hyperbolic and exponential functions to subjectively estimated probability (SP) function in terms of delay. Fig. 2 shows the SP (subjective probability estimation of obtaining delayed rewards) as a function of delay. As observed in the discounting functions, the data showed better fit for a hyperbolic, rather than an exponential function (see Table 1), supporting assumption A in the hypothesis by Sozou. Likewise, AIC_c_s for individual data were significantly smaller for hyperbolic than exponential function (t-test, *p *< 0.05), again supporting the hyperbolic decay of SP proposed by Sozou [13].

### Relations between delay/probability discount rates and SP

Spearman's correlation coefficients between *k*_*d*_*, k*_*p*_, and *k*_*sp *_(in the hyperbolic models) were presented in Table 3. A significant positive correlation was found between *k*_*d *_and *k*_*sp *_(*p *< 0.05). This result is consistent with the Sozou's hypotheses that delay discounting may be related to a hyperbolic decay of probabilities of obtaining delayed rewards as delay increases [13]. However, no significant correlation was found between probability discount rate (i.e., *k*_*p*_) and decay rate of SP (i.e., *k*_*sp*_). This finding does not contradict Sozou's theory, because Sozou's theory does not predict a significant correlation between *k*_*p *_and *k*_*sp *_[13].

### Subjective-probability discounting of delayed reward

Finally, we, as noted, estimated subjective-probability discount rate of delayed reward *k*_*spd*, _based on seven SPs obtained from SPQ (as an independent variable) and corresponding indifference points at the seven delays in the delay discounting task (as a dependent variable). In order to test assumption B', we examined the relationship between *k*_*d *_and *k*_*spd *_at the individual level. Consequently, we did not observe a significant correlation between *k*_*d *_and *k*_*spd *_(*p *> 0.1), implying that delay discounting (impulsivity in intertemporal choice) is not totally attributable to the subjective-probability discounting ("precautious uncertainty aversion" in intertemporal choice). Likewise, *k*_*spd *_did not significantly correlate with *k*_*p *_(*p *> 0.1), also supporting this conclusion.

## Discussion

### Relationship between hyperbolic delay and probability discounting

Our data on delay and probability discounting were consistent with the previous studies [1-11]. More specifically, hyperbolic functions better described both delay and probability discounting, in comparison to exponential functions [1-11]. The relationship between delay and probability discounting within subjects (i.e. correlation between *k*_*d *_and *k*_*p*_) was not significant, as reported in some previous studies [8,22]. Regarding probability discounting, the group median *k*_*p *_= 1.017 was approximately equal to 1, indicating that participants' subjective value of the probabilistic reward (in the probability discounting task) was approximately equal to a statistical expected value in the present study.

### Hyperbolic decay of subjectively estimated probability of winning delayed rewards

Interestingly, our data indicate that SP as a function of delay decays hyperbolically, rather than exponentially, which is consistent with Sozou's theory (multiple interruption/hazard rates model, see Appendix III) [13]. There was also a significant positive correlation between delay discount rate (*k*_*d*_) and subjective probability decay rate (*k*_*sp*_). To our knowledge, this study is the first to demonstrate the hyperbolic decay of the subjective probability of obtaining a delayed reward is related to hyperbolic delay discounting. Notably, previous studies [9,12], measured subjective certainty for the delayed rewards with a1-10 point rating scale and observed that the stated certainty was decreased as delay increased. Our results are qualitatively consistent with their findings.

Quantitatively, however, because (a) Patak and Reynolds' studies did not assess values of subjective probabilities [12], (b) psychological certainty is non-linearly related to a subjective-probability value [23], and the functional forms of subjective certainty for delayed rewards were not assessed in the studies, it is impossible to directly compare our present data with their data.

It is important to note that both Patak and Reynolds [12] and Sozou [13] hypothesized that the reason for delay discounting is an increase in subjective uncertainty inevitably associated with an increase in delay until receipt. This speculation logically indicates that subjects with large delay-discount rates (e.g., addicts, substance abusers and ADHDs) are dramatically risk-averse, and precautious. However, we did not observe significant relationship between subjective-probability discounting for delayed rewards (*k*_*spd*_) and delay discounting (*k*_*d*_). This finding does not support that the subjective value of a delayed reward equals the subjective-probability-discounted value of the delayed reward [13]. Psychologically speaking, impulsive subjects (i.e., subjects with greater delay discounting) may not necessarily be precautious and risk-aversive in decision over time. Therefore, it may be possible that other psychological factors than aversion to subjective uncertainty associated with delay; for instance, "impatience"; i.e., pure preference for more immediate rewards in the absence of aversion to uncertainty (also referred to as "pure time preference"), are involved in delay discounting [6]. As noted in the introduction, our results may collectively imply that a delay in intertemporal choice induces both delay discounting and an increase in subjective uncertainty (a decrease in SP) via at least two distinct psychological processes. This present hypothesis states that subjects with large delay-discount rates may have strong aversion to delay (i.e., waiting time), rather than risk-averse or precautious tendencies.

With respect to the hypebolicity of SP(D) function, it is noteworthy that another account for the observed hyperbolic decay of SP(D) is possible. Namely, if we assume that there is only a single (exponential) interruption rate *k*_*s *_but a subject has a logarithmic-time perception: *τ(D) = αln(1 + βD) *(*τ *: subjective delay as a function of objective delay, *α *and *β *are free parameters indicating psychophysical effects) in intertemporal choice [7], the resulting function of SP(D) may be (general-) hyperbolic. This can be shown as:

*SP(D) = exp(-k*_*s*_*τ(D)) = 1/(1+βD)*^*ks*α^.

Actually, a recent neuroimaging study reported that the delay length in intertemporal choice is represented in dopaminergic brain regions such as the caudate, indicating that subjects discount delayed rewards with psychological time [10,24]. Together, it can be hypothesized that when people discount delayed rewards, first, delay is psychophysically transformed into a subjective time-duration, and second, (a) delay discounting (with the subjective time-duration of delay) and (b) the estimation of SP (with the subjective time-duration of delay) occur via distinct psychological processes in a parallel manner. These possibilities should be explored in future studies, since intake/abstinence of addictive dopaminergic drugs dramatically affects time-perception, which may be associated with substance misuser's impulsivity in intertemporal choice [7,10].

Collectively, our present findings indicate that "pure time preference" (i.e., pure delay-discount rate without the effect of subjective uncertainty associated with delay) may be calculated as: [(degree of discounting of a delayed reward due to both delay *per se *and subjective uncertainty associated with delay)-(degree of discounting an uncertain reward due to uncertainty alone without delay)] = [*k*_*spd*_-*k*_*p*_], because the effects of individual differences in SP at each delay are eliminated in the estimation of *k*_*spd *_by nonlinear curve fitting of the subjective probability decay function and delays are fixed across subjects in the delay discounting task. If this parameter is positive, the subject has "pure time preference" in economics' sense (i.e., "impatience" in temporal discounting). In contrast, a conventional delay discount rate *k*_*d *_may possibly be under the effects of both pure time preference and aversion to subjective uncertainty associated with delay [6]. It should be noted that the difference between conventional delay and probability discounting rates = *k*_*d*_-*k*_*p *_cannot be utilized for this aim, because there are individual differences in the estimation of subjective probability of obtaining a delayed reward. Together, it may be recommendable for future psychopharmacological studies on discounting by substance abusers to employ the pure time preference rate (= *k*_*spd*_-*k*_*p*_) as an impatience parameter in intertemporal choice, in order to assess impatience in substance abusers and ADHDs.

Previous neuroimaging studies of intertemporal choice reported that reward-processing brain regions are activated when choosing immediate rewards [15,16]. However, these findings cannot exclude the possibility that these activations were due to higher degrees of certainty for more immediate rewards in comparison to more delayed rewards, because other neuroimaging studies demonstrated that these brain regions were also activated during decision-making under uncertainty (not intertemporal decision-making) [25]. This problem might be resolved by utilizing the pure time preference parameter proposed above in future neuroimaging studies.

### Limitation and future direction

Because the present study employed hypothetical money, it is not completely defendable that discounting behavior of real monetary rewards was reflected in the present study. Nevertheless, our results may be extendable to real rewards, because (a) discounting both hypothetical and real monetary gains follow a hyperbolic function [1-11], (b) previous studies have not observed a significant difference in the *k *parameter for hypothetical and real money rewards in a delay discounting task [26], and (c) the degrees of discounting hypothetical and real monetary gains correlated strongly [27]. Furthermore, SP at the delay of one year (about 60%) is larger than that in Patak and Reynolds' study [12] (smaller than 4 in the 1–10 point scale of which median value is (1+10)/2 = 5.5). This might be explained by (i) a confounding probabilistic factor in Patak and Reynolds' study [12]; specifically, one of the choices by participant in the delay discounting task was probabilistically honored in the study [12] and/or (ii) a hypothetical nature of the present study. Future studies should examine this point.

## Conclusion

Our present study has demonstrated that (i) subjective probability of obtaining a delayed reward (SP) decays hyperbolically, rather than exponentially, (ii) decay of SP is associated with delay discounting, but not with subjective-probability discounting, (iii) delay discounting is not completely attributable to subjective-probability discounting, (iv) the difference between subjective-probability discounting of a delayed reward and probability discounting may be a parameter of pure time preference. Future studies should examine whether the pure time preference parameter differs between healthy controls and impulsive psychiatric patients such as substance abusers [1-4,9,10] and ADHDs, who are characterized by strong temporal discounting, hypofunctioning dopaminergic systems, and impaired time-perception [28,29].

## Appendix I. Impulsivity and inconsistency in intertemporal choice

There are two distinct behavioral tendencies in intertemporal choice [6,7,14]; i.e., impulsivity and inconsistency. First, suppose the following example 1 for demonstrating impulsivity. Agent A who prefers "one apple available one year later" over "two apples available [one year plus one week] later" is more impulsive than agent B who prefers "two apples available [one year plus one week] later" over "one apple available one year later". In this example 1, most people may behave as the patient agent B. It is to be noted that both impulsive agent A and patient agent B may be rational, because, in this example 1 alone, there is no inconsistency even in impulsive agent A's behavior. Next, suppose the intertemporal choice example 2. There are two options: "one apple available now" and "two apples available one week later". In example 2, most people (who planned to choose the later option in example 1) simultaneously tend to prefer "one apple available now" over "two apples available one week later". Although the single impulsive choice of the sooner reward in the example 2 alone is not irrational, the combination of these two intertemporal choices in example 1 (choosing the later) and example 2 (choosing the sooner) is inconsistent. The reason is that the time-intervals between sooner and delayed rewards are the same (i.e., 7 days) in the two intertemporal choice problems. This inconsistency between intertemporal choice plans and actions is problematic in that even if the agent had made patient and forward-looking plans about the distant future (as in example 1), her choice plan will, as the time of executing the plan approaches to the present, be canceled and a more impulsive alternative will be chosen, as shown in example 2 (referred to as "preference reversal"). Behavioral neuroeconomic studies have demonstrated that this inconsistency may explain various problematic behaviors such as loss of self-control, a failure in formerly-planned abstinence from addictive substances and relapse. Mathematically, the inconsistency is expressed as time-dependency of a time-discount rate in hyperbolic discounting (see Appendix II).

## Appendix II. Mathematical characteristics of discount models

The degree to which a subject discounts a delayed reward (degree of discounting) is parameterized with a discount rate, defined as -(*dV*_*D*_/*dD*)/*V*_*D *_(*V*_*D *_: the subjective value of the delayed reward, *D *: the delay to the receipt of the reward) [6]. Standard economic theory assumes that the discount rate is independent of delay and the size and the sign of the delayed outcome [6]. A notable distinction between the exponential and hyperbolic discount functions exists in consistency in delay discounting; in exponential discounting, a discount rate *k*_*d *_(= -(*dV*_*D *_/*dD*)/*V > 0*, because *dV*_*D *_/*dD < 0 *when a subject discounts a delayed reward) is independent of *D *(kept constant over time), which confirms a consistency of intertemporal choice within a subject. On the contrary, in hyperbolic discounting, a discount rate defined as -(*dV*_*D*_/*dD*)/*V *= *k*_*d *_/(1+*k*_*d*_*D*) is a decreasing function of delay *D*. It is important to note that impulsivity in intertemporal choice corresponds to large discount rates, while inconsistency corresponds to a time-derivative of a discount rate = (*d/dD*) [-(*dV*_*D*_/*dD*)/*V*]. It is to be noted that "preference reversal" illustrated in Appendix I is due to a change in the discount rate (defined above) over 1 year. Specifically, devaluation of delayed rewards (two apples) over 7 days is greater for example 2 than for example 1 in Appendix I.

Because, in most discounting literature, the term "a hyperbolic discount(ing) rate" refers to a discount rate at delay *D *= 0 (i.e., *-(dV*_*D*_*/dD)/V|*_*D *= 0 _= *k*_*d*_), we followed this terminology throughout the manuscript. Note that impulsivity in intertemporal choice corresponds to a larger discount rate; while inconsistency corresponds to a more rapid decrease in a discount rate as delay increases (see Appendix I for a distinction between impulsivity and inconsistency). Furthermore, normative decision theory and microeconomics also assume that the discount rate is independent of the sign (i.e., gain or loss) and the magnitude of delayed outcomes. Behavioral economic and psychopharmacological studies have revealed that these assumptions are also violated in intertemporal choice by humans [1-11].

## Appendix III. Sozou's hypothesis

Biologically, one of the most intuitive accounts for delay discounting is that delayed rewards are discounted simply because more delayed rewards are more uncertain. Suppose that probability of obtaining delayed rewards decreases at an unknown single time-constant rate (a single interruption rate model). In this model, a subjective probability of obtaining a delayed reward (SP) is the following exponential decay function [13]:

*SP(D) = exp(-k*_*sp*_*D)*

where *k*_*sp *_indicates a decay rate of SP as delay D increases (SP = 1 when D = 0). Note that larger *k*_*sp *_corresponds to steeper decay of SP, and *k*_*sp *_is equal to delay D at which *SP = 1/e *in the single interruption rate model. Therefore, the statistical expected value of a delayed reward in the single interruption model is the following exponential discounting.

*V*_*D*_*(D) = V(0) SP(D) = V(0)exp(-k*_*sp*_*D)*,

where *k*_*sp *_is the single interruption rate. However, this single interruption rate model cannot explain empirically observed hyperbolic discounting behavior. In order to solve this problem, the evolutionary theorist Sozou proposed that there are time-independent multiple interruption rates following the exponential distribution:

*f(l) = (1/k*_*sp*_*)exp(-l/k*_*sp*_*)*

where 0 <*l *< 8 indicates each interruption rate and *k*_*sp *_is a parameter of the exponential distribution function. In this multiple interruption-rate model, when all interruption rates (*ls*) are summed by weighting with the exponential distribution, SP(D) becomes the following hyperbolic decay function:

SP=∫0∞f(l)exp⁡(−lD)dl=11+kspD,
 MathType@MTEF@5@5@+=feaafiart1ev1aaatCvAUfKttLearuWrP9MDH5MBPbIqV92AaeXatLxBI9gBaebbnrfifHhDYfgasaacH8akY=wiFfYdH8Gipec8Eeeu0xXdbba9frFj0=OqFfea0dXdd9vqai=hGuQ8kuc9pgc9s8qqaq=dirpe0xb9q8qiLsFr0=vr0=vr0dc8meaabaqaciaacaGaaeqabaqabeGadaaakeaacqWGtbWucqWGqbaucqGH9aqpdaWdXaqaaiabdAgaMjabcIcaOiabdYgaSjabcMcaPiGbcwgaLjabcIha4jabcchaWjabcIcaOiabgkHiTiabdYgaSjabdseaejabcMcaPiabdsgaKjabdYgaSbWcbaGaeGimaadabaGaeyOhIukaniabgUIiYdGccqGH9aqpdaWcaaqaaiabigdaXaqaaiabigdaXiabgUcaRiabdUgaRnaaBaaaleaacqWGZbWCcqWGWbaCaeqaaOGaemiraqeaaiabcYcaSaaa@4F31@

where *k*_*sp *_corresponds to a hyperbolic probability decay rate. Note that larger *k*_*sp *_indicates steeper decay of SP as a function of delay (SP(D)). Therefore, the statistical expected value of a delayed reward in the multiple interruption rates model is the hyperbolic delay-discounting function:

*V*_*D*_*(D) = V(0) SP(D) = V(0)/(1+k*_*sp*_*D)*,

where a (subjective) probability decay rate *k*_*sp *_equals the hyperbolic delay-discounting rate. Because Sozou's theory is only one framework which can incorporate uncertainty aversion into hyperbolic delay discounting, we utilized this framework in the present study, in order to examine the relationship between delay discounting, probability discounting, and a decay of subjective probability.

## Competing interests

The authors certify that the information listed above is complete to the best of our original research. The authors declare that they have no competing interests.

## Authors' contributions

TT is the principal researcher of the present study. KI and TH also contributed to data collection, data analysis, and experimental design. All authors read and approved the final manuscript.
